# Post-Partum Hemorrhage (PPH) as Implicated by Osler-Weber-Rendu Syndrome

**DOI:** 10.7759/cureus.43388

**Published:** 2023-08-12

**Authors:** Patrick Powers, Shanna Combs

**Affiliations:** 1 Medical Education, Burnett School of Medicine at Texas Christian University, Fort Worth, USA; 2 Obstetrics and Gynecology, Burnett School of Medicine at Texas Christian University, Fort Worth, USA

**Keywords:** obs&gy, c-section, postoperative blood loss, osler-weber-rendu, hht, hematology disorders

## Abstract

Obstetrical management of patients with genetic disorders bears its own challenges in the overall care and outcomes of the patient. Hereditary hemorrhagic telangiectasia (HHT), or Osler-Weber-Rendu Syndrome, is an inherited genetic disorder that, due to the nature of the pathophysiology of arteriovenous malformation and dysplasia, has been highly associated with an increased tendency of bleeding. This case presents a patient diagnosed with HHT prior to pregnancy who developed severe postpartum hemorrhage (PPH) after cesarean section delivery. Clinical considerations were made proactively with the knowledge and understanding of the genetic disorder, but due to unforeseen changes in the manifestation of the PPH, clinical decisions were dynamically modified in order to save the patient from exsanguination, ultimately resulting in an emergency hysterectomy and a total of 15 units of transfused packed red blood cells (pRBC). As a result, this case report serves as a preliminary recount for future research and clinical management of similar cases.

## Introduction

Hereditary hemorrhagic telangiectasia (HHT), or Osler-Weber-Rendu Syndrome, is an autosomal dominant disorder characterized by abnormal arteriovenous malformation and vascular dysplasia that has, as a result, been associated with a tendency for bleeding and hemorrhage [1-2]. The theorized pathophysiology of HHT includes mutations in Endoglin, Small Mothers Against Decapentaplegic gene family, SMAD4, and Activin, which are endothelial membrane glycoproteins serving as a co-receptor for, Transforming Growth Factor, TGF-b, necessary for angiogenesis. The Curaçao Diagnostic Criteria for HHT: -at least three of these criteria are met, it is considered to be definite HHT. -If two of the criteria are met, it is considered possible HHT. -If less than two of these criteria apply, it is unlikely to be HHT. Recurrent and spontaneous nosebleeds (epistaxis), which may be mild to severe. Multiple telangiectasias on the skin of the hands, lips, face, or inside of the nose or mouth. Telangiectasias are small red spots that disappear when pushed on. Arteriovenous malformations (AVMs) or telangiectasias in one or more of the internal organs, including the lungs, brain, liver, intestines, stomach, and spinal cord. A family history of HHT (i.e., first-degree relative such as brother, sister, parent, or child who meets these same criteria for definite HHT or has been genetically diagnosed) [3-4]. The prognosis is generally good and the telangiectasias typically manifest themselves as pseudopigmented lesions of the lips and recurrent epistaxis, but may potentially pose an increased challenge in surgical bleeding management and wound repair in an otherwise asymptomatic individual [5-7]. All this considered, the typical incidence of HHT is about 1 in 10,000 [7]. 

## Case presentation

A 36-year-old female, gravida three, para two, with a past surgical history of two uncomplicated previous low transverse cesarean sections (LTCS) at 40 weeks gestation, iron deficiency anemia, and HHT, presents for a scheduled LTCS of her third pregnancy at 39 weeks gestation. Due to the increased risk of hemorrhage, two units of packed red blood cells (pRBC) were on standby. 

The LTCS followed standard operating procedures laid out by the standard of care outlined by the American College of Obstetricians and Gynecology (ACOG). The surgery had no complications noted, with an estimated blood loss of 800 mL, which is less than the 1000 mL of blood loss for continued postpartum assessment. Bimanual massage of the uterus and baseball stitch approximation on two separate layers for anticipated primary intention wound healing was completed. Vital signs were stable.

Approximately 1 h after the LTCS, heavy vaginal bleeding with clots was noted on the fundal check. Approximately 1 h after the previous discovery of vaginal bleeding, fundal massage was repeated and 900 mL of clots was expressed. The fundus was found to be firm and 2 cm above the umbilicus. Two units of pRBCs were transfused. 

Approximately 1 h following the transfusion, a Foley catheter was placed into the vagina, through the cervix to evacuate and evaluate ongoing bleeding. Pelvic examination was difficult to assess, as the patient was in significant discomfort, but it was noted that the uterus was sitting 1 cm above the umbilicus. Nevertheless, large clots in the vagina and uterus were noted. Massive transfusion protocol (MTP) was initiated for the patient, and the decision was made to bring her to the operating room for evaluation under anesthesia. 

In the operating room, postpartum hemorrhage (PPH) was visualized and thus confirmed. Postpartum dilation and curettage and Bakri balloon placement were completed to address the atonic uterus filled with coagulated blood. The uterus was maintained at 1 cm above the umbilicus and was monitored for 30 min without rising. Bleeding was noted to be minimal. The patient was transferred to the intensive care unit (ICU) for continued care where she remained intubated.

Three hours later, the patient was noted to be bleeding again. MTP was reinitiated, with the patient receiving four more units of pRBC, two units of fresh frozen plasma (FFP), one microgram of platelets, and one microgram of cryoprecipitate. The patient was then transferred back to the operating room for an emergency hysterectomy. Upon amputation of the uterus, hemostasis was achieved, and the incisions were closed appropriately. 

 The following day, there were no reported signs of active bleeding and the patient was hemodynamically stable in no acute distress. Two additional units of pRBCs, one unit of FFP, and one microgram of platelets were transfused. In total, 15 units of pRBCs, 12 units of FFP, 3 micrograms of platelets, and 2 micrograms of cryoprecipitate were transfused in an estimated 16 h. The patient was extubated later that afternoon and continued to be hemodynamically stable with stable vital signs.

## Discussion

This case presents one of many explanations and/or causes of PPH. Although there were multiple variables affecting the management and outcome of this pregnancy, illustrated by the complex medical history pathophysiology, the authors recognize possible underlying mechanistic theories implicated in the onset of this patient’s hemorrhage, but it would be difficult to confirm the exact cause; vascular dysplasia vs AVM malformation vs epithelial weakening. This point brings to light the necessary understanding of rare genetic bleeding disorders in the preparedness to manage acute onset hemorrhage, and the mortality and morbidity associated with PPH. 

The team recognized there was a higher risk of bleeding complications in this case based on the patient’s history, having contingencies like extra blood on hand. With the promptness of the activation of the LTCS and attempts at acute stabilization of the patient, the authors were not suggesting any changes in the management of this patient. Rather, this case illustrated that perhaps the over-preparedness and low threshold for PPH management activation should be considered in patients in this demographic.

Furthermore, this case also emphasizes the acute and proper step-wise management taken in this patient to manage the PPH and acute blood loss, which ultimately would have proven to be fatal without appropriate management. The steps illustrated in this case were appropriate and effective in the overall management of this patient. In summary, the step-wise management of PPH should continue as follows: aggressive uterine massage while ensuring no retained products of conception or vaginal canal lacerations, tonic medications (methergine, carbaprost, oxytocin, misoprostol), blood products availability with transfusion for acute blood loss criteria for PPH as >1,000 mL of blood according to ACOG criteria, placement of a Bakri balloon for tamponade, B-stitch suture placement, cardinal ligament ligation, and ultimately, emergency hysterectomy. In this case, some of these steps were abbreviated or adjusted based on the rapid deterioration of this patient, as well as the nuances associated with cesarean section delivery vs vaginal delivery. 

Finally, this case does not cover the post-operative critical care management for this patient, but it should be noted that PPH of this caliber of severity does in fact require critical care time until the patient is deemed stable enough to return to the step-down management from the ICU. Management should follow the standard protocols for hypovolemic shock, acute blood loss, and hemorrhage stabilization by utilizing a complete systems approach. Of note, this patient did not require ventilatory support status post hysterectomy, but systems were in place should this patient need an escalated level of critical care management.

Overall, this case illustrates the proper stepwise management of PPH as a complication of hereditary bleeding disorders. The authors also recognize the low threshold and acute attentiveness in monitoring and acting on early signs of PPH in this demographic of patients, from blood products readily available to OR availability for Bakri placement or emergency hysterectomy. Patients with known heredity bleeding disorders are at an increased risk for PPH at baseline due to the nature of their predisposing condition [7]. The past medical history of an obstetrical patient should not be taken lightly, and should be addressed proactively, regardless of incidence and prevalence of their disease [7].

## Conclusions

Although bleeding disorders, such as Osler-Weber-Rendu, are very rare, there may be a benefit in considering readily available blood for transfusions in an obstetrical setting. Management of PPH follows with widely known MTP, nevertheless, this case illustrates the importance of placing extra consideration in preparedness for possible PPH when there is a known hereditary bleeding disorder. This case exemplifies the team’s readiness with blood products, management algorithms, and operative procedures to control the hemorrhage. These points should be carried forth with extra consideration when dealing with patients known to have a hereditary bleeding disorder. 

## References

[1] Vinay Kumar AA, Abbas AK, Aster JC (2018). Robbins Basic Pathology.

[2] Macri A, Wilson AM, Shafaat O (2021). Osler-Weber-Rendu Disease. https://www.ncbi.nlm.nih.gov/books/NBK482361/.

[3] Annalisa T, Clelia M, Antonio G, Guido C, Riccioni ME (2020). Management of gastrointestinal bleeding in Rendu-Osler disease. Rev Recent Clin Trials.

[4] Harris S, Vora NL (2018). Maternal genetic disorders in pregnancy. Obstet Gynecol Clin North Am.

[5] Inocêncio G, Braga A, Lima T (2013). Osler-Weber-Rendu syndrome during pregnancy. BMJ Case Rep.

[6] Kritharis A, Al-Samkari H, Kuter DJ (2018). Hereditary hemorrhagic telangiectasia: diagnosis and management from the hematologist’s perspective. Haematologica.

[7] Worda C, Lang I, Husslein P (2007). Hereditary hemorrhagic telangiectasia and pregnancy. Obstet Gynecol.

